# The role of personal reproductive experience on nest building in a wild bird

**DOI:** 10.1093/beheco/arag049

**Published:** 2026-04-30

**Authors:** Elisabeth G Chapman, Sophie C Edwards, Evelyn J Alexander, Susan D Healy

**Affiliations:** Centre for Biological Diversity, School of Biology, University of St Andrews, St Andrews, Fife KY16 9TH, United Kingdom; Centre for Research in Animal Behaviour, Department of Psychology, University of Exeter, Exeter EX4 4QG, United Kingdom; Centre for Biological Diversity, School of Biology, University of St Andrews, St Andrews, Fife KY16 9TH, United Kingdom; School of Psychology and Neuroscience, University of St Andrews, Fife KY16 9JP, United Kingdom; Centre for Biological Diversity, School of Biology, University of St Andrews, St Andrews, Fife KY16 9TH, United Kingdom; The Lyell Centre, Institute for Life and Earth Sciences, Heriot-Watt University, Edinburgh EH14 4AP, United Kingdom; Centre for Biological Diversity, School of Biology, University of St Andrews, St Andrews, Fife KY16 9TH, United Kingdom

**Keywords:** blue tit, breeding success, learning, nest building, nest design, reproduction

## Abstract

While much is known about the functions of avian nests in reproduction, the role of experience in nest building has often been overlooked, especially in natural populations. Increasing evidence from captive birds suggests that the environment an individual experiences affects nest-building decisions, as does reproductive experience. Here we asked whether in a wild population of blue tits *Cyanistes caeruleus*, experience affected: (i) the materials and/or amounts of material with which the birds chose to build, and/or (ii) the location in which they built. We investigated the material composition and effectiveness of nests built by female blue tits between 2016 and 2022. We found that (i) a high insulation: structure ratio was associated with higher hatching success; (ii) the rate at which nests cooled was faster the more offspring had fledged from that nest; (iii) individuals that successfully fledged at least 1 chick were more likely to reduce the amount of insulating material they put into their nest the following year than were blue tits that previously had been unsuccessful; (iv) for unsuccessful birds, changing the nest design the following year improved their fledgling success; and finally, (v) unsuccessful birds were also more likely to move to a different nestbox the following year. In sum, we find that wild blue tits used their experience of reproduction in 1 yr to change their building decisions the following year. This effect mirrors responses made by birds building in the laboratory but over a much longer time interval.

## Teaser

We investigated how the outcome of previous reproductive attempts impacted the materials and structure of nests built by wild blue tits. Nests with more insulation hatch more eggs. Birds that fledged offspring were more likely than birds that failed to fledge offspring to reduce nest insulation the following year. When previously unsuccessful birds put more insulation into their nest, their reproductive success increased. This is consistent with results found in captive birds under experimental conditions.

## Introduction

Nest building is found across a wide range of taxa, such as chimpanzees *Pan troglodytes* (Goodall 1962), paper wasps *Polistes* spp (Downing and Jeanne 1987), and 3-spined sticklebacks *Gasteroseus aculeates* (Barber et al. 2001), but it is especially common in birds (Hansell 2000; Mainwaring et al. 2014b). The functions of nests are relatively well understood. For most species, nests are used to create and maintain a suitable microclimate for eggs and chicks to develop properly (Mainwaring et al. 2014b; Gammon et al. 2020) but they can also conceal broods from predation (Hansell 1996) and be used as indicators of mate quality (Soler et al. 1998; Östlund-Nilsson and Holmlund 2003).

The functional attributes of nests have received much attention, with rather less directed to the possible roles played by the decisions made by individual builders, especially in natural populations (Breen et al. 2016). And yet, such decisions may be important. For example, several fish species adjust their nest in response to environmental conditions such as river flow (Rushbrook et al. 2010). Stickleback nests built in flowing water contain a higher proportion of spiggin (a substance secreted by males that acts as a glue), and are smaller, and more elongated than are nests built in still water, all features that may lead such nests to be more effective at coping with flowing water (Barber 2013). However, the importance of decision-making in avian nest building is less clear, potentially because avian nest building has traditionally been viewed as innate, remaining relatively unchanged throughout an individual's life (Guillette and Healy 2015; Breen et al. 2016). And yet, avian builders do seem to modify their nest design in response to temperature (eg zebra finches *Taeniopygia guttata* tend to build larger nests in cooler temperatures: Campbell et al. 2018; Edwards et al. 2020), location (eg blackbirds *Turdus merula* nests vary by latitude in the United Kingdom: Mainwaring et al. 2014a), and experience (Muth and Healy 2011; Edwards et al. 2020; Alexander et al. 2025). As most of these data come from birds that built their nests in the laboratory, here our focus is directed at the role of experience of individuals building in the wild, whether they associate the design of their nest with their reproductive success (measured as fledging chicks), and whether or not this association leads to birds adjusting their future nest design.

Location and morphology are 2 key features of nests that enable the creation of a suitable microclimate for eggs and nestlings. The location in which a nest is built exposes eggs and chicks to specific local environments, which may include predation risk, competition with conspecifics, and exposure to solar radiation. Parents make decisions on a local scale about where to build and may adjust these decisions based on prior experience. For example, collared flycatchers *Ficedula albicollis* and goldeneyes *Bucephala clangula* are more likely to build in a new nest location following an unsuccessful breeding attempt (Dow and Fredga 1983; Doligez et al. 1999). Pinyon jays *Gymorhinus cyanocephalus* build their nests lower in a tree, which leads to greater concealment of their nest, if their previous attempt suffered predation (Marzluff 1988). Similar patterns have been demonstrated in tit species, with great tits *Parus major* (Harvey et al. 1979) and marsh tits *Poecile palustris* (Wesołowski 2006) less likely to reuse a nest site following brood failure. However, moving to a different nest site is only one potential response to brood failure and may coincide with, or be avoided in favor of, changes to nest morphology.

Nest morphology can impact the success of the brood when it varies in size (Crossman et al. 2011; Windsor et al. 2013; Møller et al. 2014; Lambrechts et al. 2016a), shape (Griffith et al. 2016), or material composition (Windsor et al. 2013; Glądalski et al. 2016; Akresh et al. 2017), with each component playing a part in a nest's capacity to retain heat. By keeping eggs within suitable thermal limits heat retention in the nest by either nest size or material composition is expected to lead to more successful incubation (Webb 1987; DuRant et al. 2013). This appears to be why birds that breed in temperate environments typically incorporate insulating material, such as mammal fur and feathers, in their nests (Heenan 2013; Akresh et al. 2017) or build larger nests (Crossman et al. 2011; Mainwaring et al. 2014a). These materials, along with nest size, also help the incubating parent(s) to retain heat within the nest when the parent is absent (Diez-Méndez et al. 2021).

Zebra finches building in the laboratory show preferences for building material qualities that include material type (Bailey et al. 2014; Camacho-Alpízar et al. 2021), color (Muth and Healy 2011), and size (Muth and Healy 2014). All of these preferences may be influenced by the environment, material availability, and/or previous experience. There is increasing evidence that birds change their choices of materials and the structure of their nests based on their own experience. For example, zebra finches that built their first nest in cooler conditions used more string to build their nests than birds that built in warmer conditions (Campbell et al. 2018; Edwards et al. 2020). However, this effect was true only for birds breeding for the first time: in subsequent breeding attempts, individuals that had reared chicks successfully in their first breeding attempt used a similar amount of material to build their second nest while unsuccessful birds incorporated more material into their second nest, regardless of the ambient temperature (Edwards et al. 2020).

This work on captive zebra finches provides compelling evidence that experience changes birds’ nest-building decisions but begs the question as to whether decision-making impacts nest design in wild populations. Arguably, given that life is tougher for a wild songbird than for one living in a laboratory, the impact of reproductive decisions might be more striking. However, the time interval between reproductive events for many songbirds is often nearly a year whereas in the laboratory zebra finches this interval is just a few months. Indeed, there are multiple differences between zebra finches building in the laboratory and songbirds building in the wild. For example, laboratory zebra finches are provided with nest material whereas wild birds must choose/locate materials. Similarly, wild birds face additional challenges such as variable environmental conditions, food availability, and predation risk that birds building in the laboratory do not face. This variability faced by wild birds may make changes in nest design more subtle or difficult to identify because the nest a bird “would” build in ideal conditions may be masked by what a bird “can” build based on what is available in the environment. The “ideal nest” may also be context-dependent and influenced by factors such as environment and food availability, not only experience of the builder.

As blue tits *Cyanistes caeruleus* are commonly used to study avian reproduction in the wild, they are a useful species in which to examine nest-building decision-making (Gibb 1950; Lambrechts et al. 2010; Mainwaring 2017). Blue tits are a popular choice for monitoring wild birds due to their abundance and readiness to breed in artificial nestboxes (Serrano-Davies et al. 2017). Nest design varies by species, even within the Paridae family (Lambrechts et al. 2015, 2016b; Alambiaga et al. 2020), but also shows significant intraspecific variation. In blue tits, the female is the sole builder of the nest (Perrins 1979), which is generally constructed from moss and dried grass, and insulating materials, such as mammal fur and feathers, are used to line the nest (Mainwaring et al. 2012; Heenan 2013; Akresh et al. 2017). As blue tits build a new nest each breeding season, and in our Scottish population reproduce only once per year, we can compare building decisions made by individual females over multiple attempts. Previously successful blue tits are more likely to fledge nestlings than are naïve birds, and success of previous attempts influences the rate at which females add material to their nest (Alexander et al. 2025). However, the impacts on the amount and type of material in the nest remain unclear.

Here we aimed to determine whether reproductive experience in the form of fledging success influenced nest-building decision-making in a wild population of blue tits, specifically with regard to material composition. If reproductive experience changes female blue tit nest-building decision-making in ways similar to the changes made by experienced male zebra finches in the laboratory (Edwards et al. 2020), we expected that blue tits whose prior breeding attempt failed to produce fledglings would be more likely than previously successful blue tits to increase the amount of insulation included in their subsequent nest.

In addition, unlike birds in the laboratory, blue tits building in the wild can make decisions not just about the material composition of their nest but also where to build their nest. As successful birds seem to repeat their decisions when building their subsequent nest, we also expected that previously unsuccessful blue tits would be more likely to move nest location, a decision made by unsuccessful goldeneye and collared flycatchers (Dow and Fredga 1983; Doligez et al. 1999).

To address these questions, we compared nests built by individual blue tits building in nestboxes around St Andrews, Fife, Scotland over 2 consecutive years between 2016 and 2022. Nests were monitored throughout their building stages with the female builder and male partner individually identified. Number and timing of eggs, hatchlings and fledglings were recorded for individuals over multiple years. At the end of the breeding season, we estimated the ability of the nest to retain heat by calculating the cooling coefficient (McGowan et al. 2004) and deconstructed nests to determine their material composition. We expected unsuccessful birds to be more likely than successful birds to (i) increase the amount of insulation they put into their nest, and (ii) move to a different nestbox.

## Materials and methods

### Study population

We collected data between 2016 and 2022 from 81 to 88 nestboxes located in the St Andrews Botanic Gardens and the University of St Andrews grounds in St Andrews, Scotland (2016: *n* = 81, 2017: *n* = 81, 2018: *n* = 88, 2019: *n* = 87, 2021: *n* = 87, 2022: *n* = 86). Schwegler model 1B nestboxes (internal chamber 170 mm width × 180 mm depth; 26 mm entrance hole) were hung on trees in a variety of semi-urban habitats, orientations, and heights (1 to 2.5 m from the ground). Many individuals in this population are fitted with BTO rings as well as unique combinations of color rings to allow individual identification and monitoring over multiple years. Data were not collected in the 2020 breeding season due to the Covid-19 pandemic.

### Field observations

Each year, all nestboxes in the study area were monitored every 4 d from the beginning of March, and every 2 d after building material (grass or moss) had been found in at least 1 nestbox. Once we had observed the presence of lining (feathers or mammal fur), we checked the nests for eggs by gently feeling inside the nest cup and counting eggs. If more than 1 egg was present, we assumed a laying rate of 1 egg per day (Perrins 1979) and recorded the date on which we assumed egg laying to have begun. Incubation initiation was determined by the presence of warm eggs, or if the female was seen sitting on at least 4 eggs (Edwards 2019). Once incubation had begun for a nest, we reduced the checks to every 4 d. 5 to 10 d after incubation began, female adults were caught on the nest and fitted with a BTO ring and a unique combination of color rings. Individuals were sexed by the presence of a brood patch, and aged based on their post-juvenile molt (Jenni and Winkler 1994). Chicks were ringed prior to fledging, and adult males were caught, identified, and ringed during the rearing period. Once broods had fledged, around 18 d after hatching, we counted and identified any dead chicks. All chicks approaching fledging age that we did not find dead we assumed to have fledged successfully. If eggs or chicks went missing prior to fledging, we assumed they had been taken by a predator.

#### Ethical note

This study was approved by the University of St Andrews Animal Welfare and Ethics Committee. All bird ringing was carried out under BTO ringing license.

### Nest processing

After chicks had fledged, we removed nests from the nestboxes and froze them at −80 °C for at least 4 d to kill any invertebrates. We then either air-dried them in a greenhouse for at least 1 wk (2021 to 2022 nests) or for 48 h in an oven at low temperature (2016 to 2019 nests, Edwards 2019).

We measured the insulative capacity of each nest by assessing the rate at which it cooled. We used the cooling coefficients following methods developed from McGowan et al. (2004). Two temperature loggers (iButtons; Model DS1922L, accuracy ± 0.5°C) were programmed to record temperature every 20 s and heated to 80 °C in a water bath. One iButton was then placed in the nest cup and the other, a control iButton, placed on a plastic surface at equivalent height outside the nest. Both iButtons were left for 30 min, during which time they both cooled to room temperature (18 to 22 °C). This was repeated twice for each nest, to obtain an average. The cooling coefficient was determined using Equation (1) where *B* and *C* are fitted constants. *B* is the initial gradient between the iButton and the ambient temperature and *C* is the rate of cooling of the iButton (McGowan et al. 2004; Edwards 2019). The cooling coefficient was calculated as the value of *C* for the nest iButton minus the value of *C* for the control iButton. A higher cooling coefficient indicated that the nest iButton had cooled down more slowly, and that the nest was better insulated.


(1) $$\mathrm{iButton}\;\mathrm{Temp}.\;=\mathrm{Ambient}\;\mathrm{Temp}.+[B_{\exp}\;(-C\times \mathrm{Time})]$$

Intact nests were weighed to the nearest 0.01 g using an FGL-made, other (containing dead chicks, eggshells, insects, and unidentifiable material), and fine material (dust left over at the end of deconstruction). Each category of material was then weighed. We excluded “other” and “fine” materials from all analyses because “other” contains mostly materials that were not intentionally incorporated into the nest and “fine” was made up of small remnants of materials, the origin of which we could not determine. When we found dead chicks during a deconstruction, we adjusted the number of hatchlings and fledglings for that nest accordingly. For the purposes of analyses, “insulation” was the combination of the mass/percentage of mammal fur/hair and feathers and “structural” was the combination of the mass/percentage of moss and grass.

### Data analysis

We used R version 4.5.1 (R Core Team 2025) to analyze the data, using lme4 (Bates et al. 2015) to create models and ggplot2 (Wickham 2016) to create figures. We created a series of linear mixed models (LMMs) and generalized LMMs (GLMMs) to address our questions. Final models were selected by inspection of residuals using the DHARMa package (Hartig 2022) and comparison of Akaike Information Criterion (AIC) values. We performed Type II likelihood-ratio *χ*^2^ tests using the car package (Fox and Weisberg 2018) on finalized LMMs to determine significance between levels. For significant models looking at the effect of previous success (binary) on nest materials, we ran post hoc tests using the emmeans package (Lenth et al. 2025) to determine whether each level significantly differed from zero (Tables S2, S5). Model selection criteria, AIC values, and model summary outputs are presented in the Supplementary material.

#### Effect of personal reproductive experience on nest design

We used data from 111 blue tit nests built by females that had built a nest in the study area in the previous year. For each nest, we identified the female builder, and the success of her previous nest by the number of successful fledglings.

We calculated differences between a female's nests by subtracting the mass (g) and percentage (%) of each material from the current nest from that of her previous nest. A positive difference indicated that more of a given material had been included than had been included in their previous nest. A negative difference indicated that less of a given material had been included than in the previous nest.

We first asked how previous success impacted nest design in the next attempt. To do this we looked at material type based on function (insulation, structural) as well as origin (mammal fur/hair, feathers, grass, moss) for both mass (g) and percentage (%) of total nest. Previous fledgling number was zero-inflated with a bimodal distribution, so we chose to model the effect of having any fledglings (fledgling presence) separately from the effect of number of fledglings (fledgling number). We used LMMs to look at the effect of previous fledgling success (binary predictor variable: 1 or more chicks fledged [success]/zero chicks fledged [failed]) on the change in nest characteristic. We then repeated these analyses with only those females that had produced at least 1 fledgling from her previous nest (*n* = 87) to ask how previous fledgling *number* influenced subsequent nest design. To account for multiple measures of individual females and within-year effects, both female ID and year were fitted as random effects but were removed from models if they did not explain sufficient variation (Tables S1, S3, S4, S6).

#### Effects of differences in nest design

We then used all the available nest data for completed nests (nests were considered completed if they contained at least 1 egg; *n* = 335 nests) to determine the effects of differences in nest design. Here we used both the number of eggs that hatched and the number of hatchlings that fledged as proxies for reproductive success. Including the number of hatchlings allowed us to focus on effectiveness of the nest during incubation rather than other aspects of parenting that are more likely to have affected the number of fledglings eg parental provisioning (Mutzel et al. 2013).

Both the number of eggs that hatched and the number of hatchlings that fledged were zero-inflated with a bimodal distribution so we used hurdle models using the pscl package (Zeileis et al. 2008; Jackman 2020) to determine how nest design impacted both numbers. Insulation: structure ratio was calculated as the mass of insulation divided by the mass of structural material; a high insulation: structure ratio indicates proportionally more insulation in the nest. These models assessed the effect of insulation: structure ratio of the nest on the presence and number of eggs hatched/chicks fledged in 2 steps. These models included the number of hatchlings or fledglings as the response variable with insulation: structure ratio as the predictor.

To determine the insulative capacity of nests, we again ran hurdle models to determine the effects of cooling coefficient on hatchling and fledgling success. We also used an LMM to verify the effectiveness of using cooling coefficients in this way. This model included cooling rate as the response variable, with insulation (g) as the predictor variable (insulation material content should positively predict cooling rate). Random effects of female ID and year were included (see Table S9).

We then asked whether the changes made by a female lead to an increase in her success at fledging her hatchlings. Here we focused again on the nests for which we had data on the female's previous attempt (*n* = 111). We calculated a difference in success by subtracting the previous number of fledglings from the current number of fledglings. A positive difference indicated that fledging success of a given female increased between years. A negative difference indicated that fledging success of a given female decreased between years. We ran an LMM with the difference in insulation (g) as the predictor variable and difference in fledgling number as the response variable. Female ID and year were included as random effects and female ID was removed from the final model as it did not explain sufficient variation (see Table S8). This was then repeated on subsets of data that separated females that had previously failed to fledge any chicks with those that had previously fledged at least 1 chick (see Table S8).

#### Effect of personal reproductive experience on nestbox choice

We used binomial GLMMs (binomial GLMM, “logit” link) to determine the effect of fledging success on the likelihood that individuals moved to a different nestbox for the next breeding attempt. To do this, we categorized individuals as having built in a different nestbox or returned to their previous nestbox in their subsequent attempt. Again, we looked at previous fledging success in 2 parts: fledgling presence and fledgling number. These models included previous fledging success (either presence or number) as the predictor variables and whether or not the female moved nestbox as the response variable. Female ID and year were included as random effects but removed from final models if they did not explain variation (see Table S10). Instances where the nestbox occupied by the female in the previous year had been removed, or changed location, were removed from analysis as the individuals had no choice but to move nestbox (*n* = 3) giving a sample size of 108 nests for these analyses.

We also ran a binomial GLMM to determine whether moving nestbox was associated with mating with a different male. This model included a binary predictor variable (mated with a different male: yes/no) and a binary response variable (moved nestbox: yes/no) with female ID and year included as random effects and removed as before (see Table S10). This could only be done for cases where both the male and the female from 2 subsequent attempts had been caught/identified (*n* = 47).

A further LMM with change in fledgling number between years as the response variable and whether the female moved nestbox (yes/no) as the binary predictor variable was used to determine if there was a relationship between moving nestbox and reproductive success the following year for the individual. Again, female ID and year were included as random effects in this model and removed if they did not explain variation (see Table S10).

## Results

### Effect of previous fledging success on nest design

#### Insulation material

**Figure 1:**
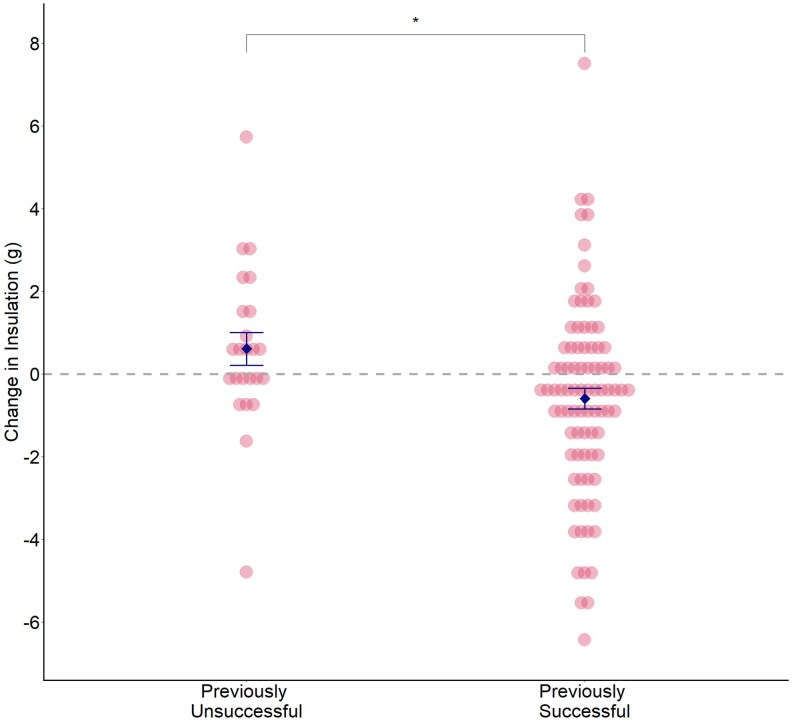
Mean differences in mass (g) of insulating material (mammal fur and feathers combined) between nests built by females that had failed to fledge any chicks in their last attempt (previously unsuccessful) and females that had previously fledged at least 1 chick (previously successful). Pink dots represent data for individual nests; the blue diamonds show group means ± SE (previously unsuccessful: *n* = 25, previously successful: *n* = 86; *Significance level of *P* < 0.05).

Females that had fledged chicks from their previous nest were more likely to reduce the mass of insulation incorporated into their subsequent nest than were females that had failed to fledge chicks from their previous nest (LMM: $\chi_{1}^{2}$ = 6.48, *P* = 0.011; Fig. 1).

When we separated insulation into its constituent parts (mammal fur and hair/feathers), we found that these differences occurred in mammal fur rather than in feathers. Females that had fledged chicks previously were more likely than unsuccessful females to decrease the mass (LMM: $\chi_{1}^{2}$ = 6.42, *P* = 0.011) and percentage (LMM: $\chi_{1}^{2}$ = 3.91, *P* = 0.048) of mammal fur and hair in their subsequent nest. However, there was no difference in the mass (LMM: $\chi_{1}^{2}$ = 0.15, *P* = 0.702) or percentage (LMM: $\chi_{1}^{2}$ = 0.845, *P* = 0.358) of feathers they incorporated. In addition, there was no effect of the “number” of chicks she had fledged on her incorporation of insulation in the subsequent nest. This was true for both mass (LMM: $\chi_{1}^{2}$ = 0.10, *P* = 0.746) and percentage (LMM: $\chi_{1}^{2}$ = 1.02, *P* = 0.311) of insulation.

#### Structural material

**Figure 2:**
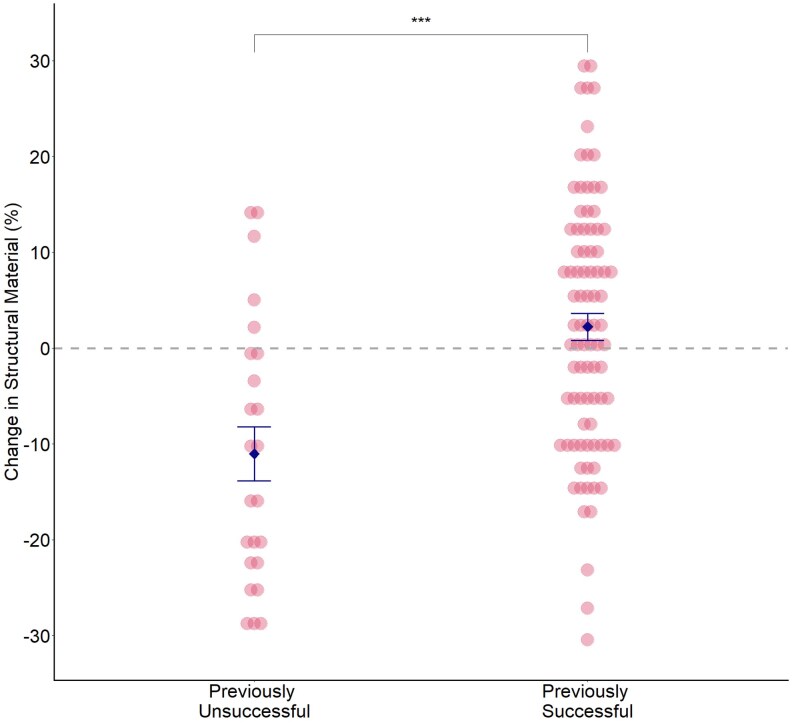
Mean differences in mass percentage (%) of nest made up of structural material between nests built by females who had failed to fledge any chicks in their last attempt (previously unsuccessful) and females who had previously fledged at least 1 chick (previously successful). Pink dots represent data for individual nests; the blue diamonds show the group means ± SE (previously unsuccessful: *n* = 25, previously successful: *n* = 86; ***Level of significance at *P* < 0.001).

There was no difference in the mass of structural material incorporated based on previous fledgling success (LMM: $\chi_{1}^{2}$ = 1.10, *P* = 0.295). However, females that had not fledged chicks from their previous nest were more likely to subsequently incorporate “proportionally” less structural material into their subsequent nest and females that had fledged chicks were more likely to increase the percentage of structural material they included into their nest (LMM: $\chi_{1}^{2}$ = 16.8, *P* < 0.001; Fig. 2). There was no effect of the “number” of chicks a female had fledged on the incorporation of structural material (mass: $\chi_{1}^{2}$ = 0.68, *P* = 0.411; percentage: $\chi_{1}^{2}$ = 0.02, *P* = 0.896).

When we separated structural material into its constituent parts (moss/grass), we found no difference between the amount of moss (mass: LMM: $\chi_{1}^{2}$ = 0.59, *P* = 0.441) in the nests of previously successful and previously unsuccessful females. However, in their subsequent breeding attempt previously successful females included a greater percentage of grass than they had previously (LMM: $\chi_{1}^{2}$ = 4.9, *P* = 0.027).

#### Effect of nest structure on reproductive success

**Figure 3:**
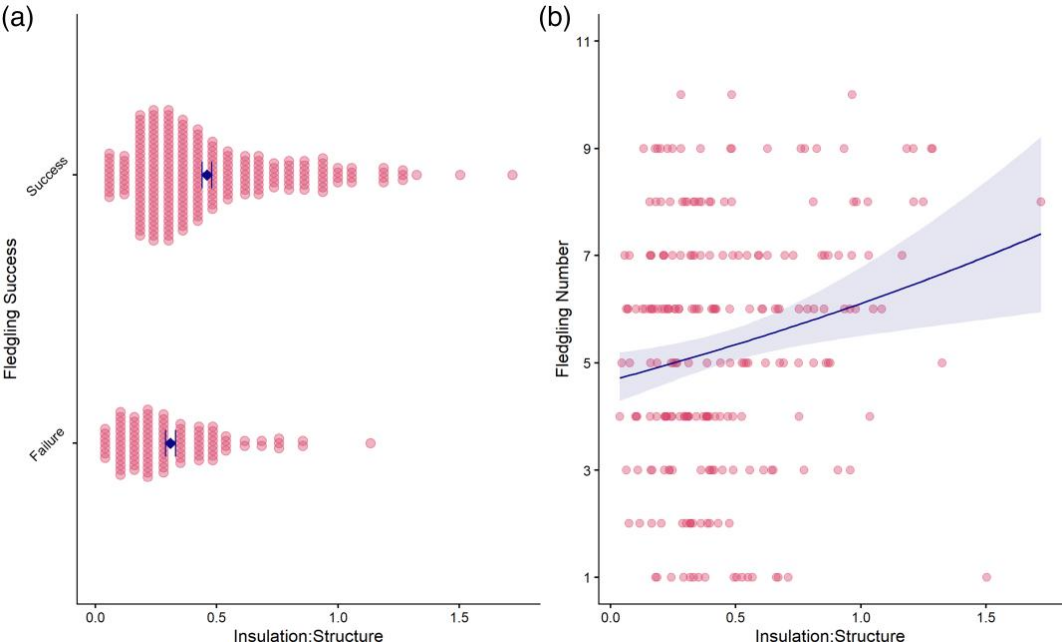
Relationship between insulation: structure ratio and fledgling success. a) Whether any chicks survived to fledge (success) or all chicks died (failure). Pink dots represent data for individual nests; the blue diamonds show the group means ± SE. b) The number of chicks that survived to fledge in those nests where at least 1 chick did so (*n* = 235). Pink dots represent data for individual nests; the blue line indicates the model prediction ± SE.

Eggs were more likely to hatch at all, and more of them to hatch, in nests that had a higher insulation: structure ratio (hurdle: estimate = 1.89, *P* = 0.008; number of eggs: estimate = 0.22, *P* = 0.005). This relationship also carried through to fledging success. Chicks were more likely to survive, and more chicks were likely to survive, in nests that contained proportionally more insulation (hurdle: estimate = 2.32, *P* < 0.001; number of chicks surviving to fledge: estimate = 0.27, *P* = 0.002; Fig. 3a and b).

**Figure 4:**
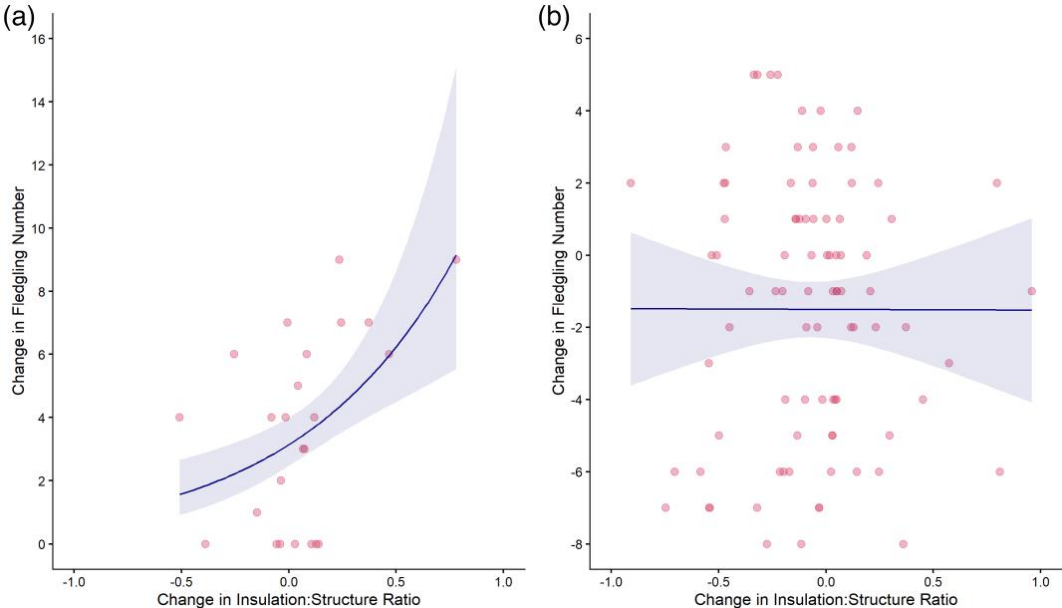
Relationship between changes in insulation: structure ratio between 2 consecutive attempts and the change in fledgling number between these 2 attempts. a) Nests built by females that had previously failed to produce live fledglings (*n* = 24). b) Nests built by females that had previously had at least 1 chick survive to fledge (*n* = 87). Pink dots represent data for individual nests and the blue lines indicate model predictions ± SE.

Changes in nest design tended to lead to changes in fledging success for all birds, although this effect was not significant (LMM: $\chi_{1}^{2}$ = 3.21, *P* = 0.07). However, when we looked only at females that had previously failed to fledge any chicks, increasing the insulation: structure ratio improved her fledging success (LMM: $\chi_{1}^{2}$ = 14.55, *P* < 0.001; Fig. 4a), an effect we did not see for females that had previously fledged chicks (LMM: $\chi_{1}^{2}$ = 0.003, *P* = 0.9596; Fig. 4b).

#### Effect of reproductive success on heat retention

We found that both hatchling success and fledgling success increased the faster a nest cooled. Fewer eggs had hatched, and fewer chicks survived to fledging in the nests that retained heat better (hurdle: estimate = −24.91, *P* < 0.001; number of fledglings: estimate = −27.35, *P* < 0.001). Furthermore, the more insulation material a nest contained was not associated with better heat retention (mass: LMM: $\chi_{1}^{2}$ = 2.13, *P* = 0.145; percentage: LMM: $\chi_{1}^{2}$ = <0.01, *P* = 0.959).

### Effect of personal experience on nestbox choice

**Figure 5:**
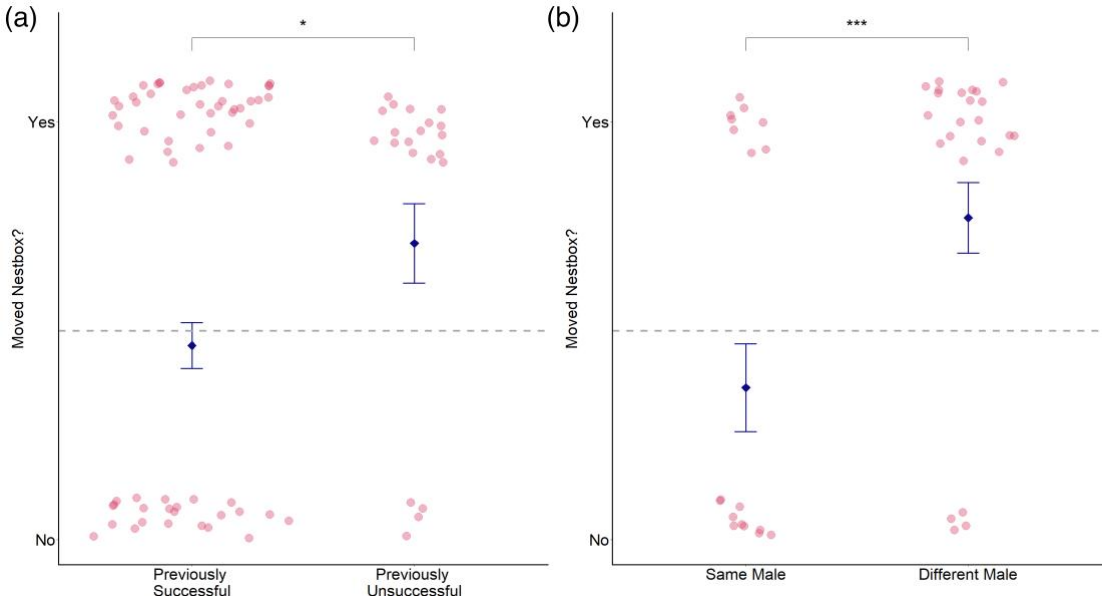
a) Relationship between previous success and likelihood of moving to a different nestbox. b) Relationship between mating with a different male and likelihood of moving to a different nestbox. Pink dots represent values data for individual nests; the blue diamonds represent group means ± SE (***P < 0.001; *P < 0.1).

In total, around 52% of females moved nestbox between 2 consecutive attempts. However, 71% of previously unsuccessful females and only 46% of previously successful females moved to a different nestbox. Previously unsuccessful birds tended to be more likely to move to a different nestbox in their subsequent breeding attempt than were previously successful birds (GLMM: $\chi_{1}^{2}$ = 3.80, *P* = 0.051; Fig. 5a). However, there was no relationship between the likelihood of moving nestbox and the “number” of chicks they had previously fledged (GLMM: $\chi_{1}^{2}$ = 0.50, *P* = 0.479).

Individuals that bred in a different nestbox moved highly variable distances, which ranged between 15.71 and 457.55 m (mean = 92.86 m, SD = 89.20 m). Females that moved nestbox were also more likely to mate with a different male (GLMM: $\chi_{1}^{2}$ = 18.77, *P* < 0.001; Fig. 5b). However, of the 26 instances of females remating, only 2 (7.7%) were the result of “divorce.” For the remaining 24 instances, we assumed that the male had died as he was not seen again in the population.

The females that moved to a different nestbox in the following year were not more likely to improve their fledgling success (LMM: $\chi_{1}^{2}$ = 0.47, *P* = 0.495).

## Discussion

Contrary to our expectations, female blue tits that successfully fledged chicks in their previous attempt were more likely than previously unsuccessful females to change their nest design in their subsequent attempt by reducing the amount of insulation and including proportionally more structural material (Figs. 1 and 2). In contrast, birds that had failed to fledge any chicks did not change the absolute amount of insulation they included into their nest the following year, but they were more likely to increase the percentage of insulation (Fig. 1). Hatching and fledging success were higher when a nest contained both absolutely and relatively more insulation (Fig. 3) but changes in insulation only led to increased fledgling success for those females that had previously failed to fledge any chicks (Fig. 4). Taken together, these data suggest that female blue tits learn and implement effectiveness of nest design to improve their reproductive success in subsequent breeding attempts. Our data are consistent with the responses to breeding success of laboratory zebra finches (Bailey et al. 2014; Edwards et al. 2020) and show that for wild birds too, nest building involves learning the effectiveness of material type and nest design and implementing that knowledge when building subsequent nests.

In blue tits, and other avian species, experienced birds fledge more chicks than do naïve individuals (Perrins 1970; Limmer and Becker 2010; Alexander et al. 2025; Healy 2025). Our data suggest, however, that the kind of experience itself modifies the decisions made by the nest builder: unsuccessful birds increased the insulation in their nest while successful birds effectively decreased the insulation in their nest. Both these responses suggest that the birds can associate their success (or lack of) with the warmth of the nest that they build and, possibly the effort they expended in incubation. The data we present conflicts with the consistency in the composition of the nest cup built by Northern house wrens *Troglodytes aedon*, which was repeatable within individuals between years (Carr et al. 2024). Similarly, nest size (measured as nest depth) in a different population of blue tits was consistent within females (O’Neill et al. 2018). The differences between this earlier work and our data may be due to our deconstructions of the entire nest rather than simply measuring the depth of nest in the nestbox. While nest size may be repeatable within females, it appears that the composition of that size (the relative amounts of different materials) is flexible and depends, at least partly, on the female's experience.

However, despite finding that both hatching and fledging success increased with insulation, we were surprised to find that nests that cooled down more slowly were the nests that had resulted in less hatching success. Cooling coefficients have been used as a proxy for the ability to the nest to retain heat during incubation (McGowan et al. 2004; Mainwaring et al. 2012), and it is assumed that a high cooling coefficient indicates effective insulation and slower cooling. However, measurements on cooling coefficients have typically been made at the end of the breeding season and therefore may give a misleading representation of the insulative properties of the nest during incubation. It seems plausible that the more chicks produced in a nest leads to a greater compression of the nest and cup as those chicks grow and move about the nest. This compression is likely to lead to a lower cooling coefficient. Cruz et al. (2016) found that the insulative capacity of great tit nests decreased from incubation to post-fledging, but because the 2 measures were correlated, argued that for great tit nests, at least, it was possible to assess insulative capacity from nests measured post-fledging (Cruz et al. 2016). In contrast, our nests that had few, or no, chicks grow in them retained their insulative capacity, perhaps because they were more intact at the end of the breeding season, and amount of insulation material in a nest was not related to a slower cooling rate. Taken together these results suggest that cooling coefficients measured post-fledging is not always an accurate measure of the heat retention of the nest during incubation and might, therefore, be treated with caution.

We also found that females that had experienced a failed breeding attempt were more likely to build in a different nestbox in their subsequent breeding attempt. Similarly, collared flycatchers are more likely to disperse following brood failure (Doligez et al. 1999) while pinyon jays *Gymnorhinus cyanocephalus* (Marzluff 1988) and Northern flickers *Colaptes auratus* (Fisher and Wiebe 2006), individuals are more likely to change nest location following nest predation. In our population, predation rates were relatively low: in the 6 yrs of data we analyzed, there were just 10 instances of clutch/brood predation. It seems unlikely therefore that a female's decision to move nestbox in this population was due to predation risk. Rather, the increase in likelihood of deciding to build in a different nestbox may have been birds attempting to improve the quality of their habitat or partner, or to reduce competition. We do not have the data to distinguish among these possibilities.

These results provide evidence that experience plays a role in the decisions blue tits make when selecting nest materials. However, there are other potential causes of variation that should be considered. For example, phenology of reproduction may interact with the nest design. In temperate regions, individuals that begin to lay eggs earlier in the season may require better insulated nests than those that delay egg laying. For example, Great tits build larger nests built at colder temperatures, with nest size correlated with the maximum daily temperature experienced around 2 to 3 wk prior to egg-laying (Mazgajski et al. 2025). It may also be that birds face trade-offs when selecting nest materials and their choice of nest materials may also depend on the timing of building and egg-laying. This may not always be in the direction of increased warming as although insulation materials help to maintain temperatures around the eggs and chicks it can also increase the risk of nestling hyperthermia (Mertens 1977; Lombardo 1995). Adults may need to continue to be flexible with their nest materials even after eggs hatch as barn swallow *Hirundo rustica* parents remove feathers from the nest post-hatching once their chicks become able to thermoregulate (Møller 1987). Great tits and blue tits also seem to adjust their nest design both prior to, and post, egg-laying (Britt and Deeming 2011; Harnist et al. 2020; Mazgajski et al. 2025).

In sum, we show that wild blue tits that experienced brood failure increased their reproductive success in their subsequent attempt by changing their nest design. Females that experienced brood failure were also more likely to move to a different nestbox the following year, potentially associated with an attempt to mate with a higher quality male. A key question that our data raise is how both wild blue tits and laboratory zebra finches know when their nest will enable them to provide a suitable thermal microclimate for hatching their eggs.

## Supplementary Material

arag049_Supplementary_Data

## Data Availability

Analyses reported in this article can be reproduced by using the data provided by Chapman et al. (2026).
