# Supplementary material for: The role of personal reproductive experience on nest building in a wild bird
Source: Behav Ecol. 2026 Apr 30;37(4):arag049. doi: 10.1093/beheco/arag049 (PMC13202215; doi:10.1093/beheco/arag049)
Supplement: arag049_Supplementary_Data [file arag049_supplementary_data.docx]

**Table S1.** Model structure, selection process and summary output of general linear mixed models looking at the effect of previous success (binary) on the difference (Δ) in amount (g) and proportion (%) of insulation material (feathers and mammal hair) included in nests between two consecutive attempts. All model output values are given to 3 decimal places. (Significance codes: < 0.05 ‘*’). Previous success was coded as a factor (Success/Fail).

| **MODEL NUMBER** | **RESPONSE VARIABLE** | **MAXIMAL MODEL** | **FINAL MODEL** | | **MODEL SELECTION** | **FINAL MODEL SUMMARY** | | | | | |
| --- | --- | --- | --- | --- | --- | --- | --- | --- | --- | --- | --- |
|  |  |  |  |  |  |  | **Estimate** | **Std Error** | **df** | **T value** | **P value** |
| Mod1b | Δ Insulation (g) | PreviousSuccess + (1\|MotherID) + (1\|Year) | PreviousSuccess + (1\|Year) | | (1\|MotherID) explained 0 variance (Maximal model AIC: 501.2, Final model AIC: 499.2) | Intercept | 0.713 | 0.560 | 10.216 | 1.189 | 0.262 |
|  |  |  |  |  |  | Previous Success | -1.326 | 0.521 | 108.836 | -2.546 | 0.012* |
| Mod3b | Δ Insulation (%) | PreviousSuccess + (1\|MotherID) + (1\|Year) | PreviousSuccess + (1\|Year) | | (1\|MotherID) explained 0 variance (Maximal model AIC: 855.7, Final model AIC: 853.7) | Intercept | 1.277 | 3.639 | 6.508 | 0.351 | 0.737 |
|  |  |  |  |  |  | Previous Success | -4.001 | 2.637 | 107.960 | -1.518 | 0.132 |
| Mod5b | Δ Mammal hair (g) | PreviousSuccess + (1\|MotherID) + (1\|Year) | PreviousSuccess + (1\|Year) | | (1\|MotherID) explained 0 variance (Maximal model AIC: 487.1, Final model AIC: 485.1) | Intercept | 0.521 | 0.684 | 6.399 | 0.762 | 0.483 |
|  |  |  |  |  |  | Previous Success | -1.231 | 0.486 | 107.896 | -2.535 | 0.013* |
| Mod7b | Δ Mammal hair (%) | PreviousSuccess + (1\|MotherID) + (1\|Year) | PreviousSuccess + (1\|Year) | | (1\|MotherID) explained 0 variance (Maximal model AIC: 842.9, Final model AIC: 840.9) | Intercept | 1.650 | 3.725 | 5.721 | 0.443 | 0.674 |
|  |  |  |  |  |  | Previous Success | -4.909 | 2.481 | 107.631 | -1.978 | 0.050 |
| Mod9a | Δ Feathers (g) | PreviousSuccess + (1\|MotherID) + (1\|Year) | | PreviousSuccess + (1\|Year) | Model fit with (1\|MotherID) and it explained some variance (0.018, sd 0.133). However, removing (1\|MotherID) does not influence interpretation of results. Examination of AIC suggests that removing (1\|MotherID) is the better model (AIC maximal model: 332.5, final model 330.5). | Intercept | 0.191 | 0.254 | 13.720 | 0.753 | 0.464 |
|  |  |  |  |  |  | Previous Success | -0.097 | 0.240 | 108.972 | -0.402 | 0.688 |
| Mod11b | Δ Feathers (%) | PreviousSuccess + (1\|MotherID) + (1\|Year) | PreviousSuccess + (1\|Year) | | (1\|MotherID) explained 0 variance (Maximal model AIC: 674.4, Final model AIC: 672.4) | Intercept | -0.517 | 1.168 | 15.879 | -1.442 | 0.664 |
|  |  |  |  |  |  | Previous Success | 1.059 | 1.152 | 108.642 | 0.919 | 0.360 |

**Table S2.** For significant models predicting a relationship between previous success and nest materials, we used the “emmeans” package to determine if each factor level was significantly different from zero (no change) between two years.

| **Model** | **Previous Success Level** | **emmean** | **SE** | **df** | **Lower CL** | **Upper CL** | **T ratio** | **P value** |
| --- | --- | --- | --- | --- | --- | --- | --- | --- |
| Mod1b | Fail | 0.713 | 0.605 | 9.92 | -0.637 | 2.062 | 1.178 | 0.266 |
|  | Success | -0.613 | 0.449 | 3.33 | -1.964 | 0.738 | -1.366 | 0.257 |
| Mod5b | Fail | 0.521 | 0.686 | 6.27 | -1.140 | 2.18 | 0.759 | 0.475 |
|  | Success | -0.710 | 0.574 | 3.17 | -2.48 | 1.06 | -1.238 | 0.300 |

**Table S3.** Model structure, selection process, and summary output of general linear mixed models looking at the effect of previous success (count of previous fledglings) on the difference (Δ) in amount (g) and proportion (%) of insulation (feathers and mammal hair) included in nests between two consecutive attempts. All model output values are given to 3 decimal places. (Significance codes: < 0.05 ‘*’). Previous fledge was coded as numeric.

| **MODEL NUMBER** | **RESPONSE VARIABLE** | **MAXIMAL MODEL** | **FINAL MODEL** | **MODEL SELECTION** | **FINAL MODEL SUMMARY** | | | | | |
| --- | --- | --- | --- | --- | --- | --- | --- | --- | --- | --- |
|  |  |  |  |  |  | **Estimate** | **Std Error** | **df** | **T value** | **P value** |
| Mod2b | Δ Insulation (g) | PreviousFledge + (1\|MotherID) + (1\|Year) | PreviousFledge + (1\|Year) | (1\|MotherID) explained 0 variance (Maximal model AIC: 501.2, Final model AIC: 403.42) | Intercept | -0.802 | 0.745 | 20.584 | -1.077 | 0.294 |
|  |  |  |  |  | Previous Fledge | 0.036 | 0.110 | 84.686 | 0.323 | 0.747 |
| Mod4b | Δ Insulation (%) | PreviousFledge + (1\|MotherID) + (1\|Year) | PreviousFledge + (1\|Year) | (1\|MotherID) explained 0 variance (Maximal model AIC: 680.2, Final model AIC: 678.2) | Intercept | -5.770 | 4.272 | 11.034 | -1.351 | 0.204 |
|  |  |  |  |  | Previous Fledge | 0.556 | 0.550 | 83.737 | 1.012 | 0.314 |
| Mod6b | Δ Mammal hair (g) | PreviousFledge + (1\|MotherID) + (1\|Year) | PreviousFledge + (1\|Year) | (1\|Mother ID) explained very little variance (7.002e-06, sd = 0.003) (Maximal model AIC: 393.09, Final model AIC: 391.1) | Intercept | -0.367 | 0.819 | 9.340 | -0.447 | 0.664 |
|  |  |  |  |  | Previous Fledge | -0.061 | 0.101 | 83.560 | -0.598 | 0.552 |
| Mod8b | Δ Mammal hair (%) | PreviousFledge + (1\|MotherID) + (1\|Year) | PreviousFledge + (1\|Year) | (1\|MotherID) explained 0 variance (Maximal model AIC: 670.8, Final model AIC: 688.8) | Intercept | -3.809 | 4.383 | 8.577 | -0.869 | 0.409 |
|  |  |  |  |  | Previous Fledge | 0.107 | 0.518 | 83.352 | 0.207 | 0.837 |
| Mod10a | Δ Feathers (g) | PreviousFledge + (1\|MotherID) + (1\|Year) | PreviousFledge + (1\|MotherID) + (1\|Year) | Model fit with (1\|MotherID) explained some variance (0.135, sd = 0.368). Examination of AIC values suggest retaining (1\|MotherID) is the better model (Maximal model AIC: 270.8, removing (1\|MotherID) AIC: 272.7). However, removing (1\|MotherID) does not influence interpretation of results. | Intercept | -0.382 | 0.349 | 18.972 | -1.096 | 0.287 |
|  |  |  |  |  | Previous Fledge | 0.088 | 0.050 | 84.586 | 1.749 | 0.084 |
| Mod12b | Δ Feathers (%) | PreviousFledge + (1\|MotherID) + (1\|Year) | PreviousFledge + (1\|Year) | (1\|MotherID) explained 0 variance (Maximal model AIC: 528.6, Final model AIC: 526.6) | Intercept | -1.876 | 1.644 | 13.926 | -1.141 | 0.273 |
|  |  |  |  |  | Previous Fledge | 0.434 | 0.226 | 84.12 | 1.922 | 0.058 |

**Table S4.** Model structure, selection process, and summary output of general linear mixed models looking at the effect of previous success (binary) on the difference (Δ) in amount (g) and proportion (%) of structural material (moss and grass) included in nests between two consecutive attempts. All model output values are given to 3 decimal places. (Significance codes: < 0.05 ‘*’, < 0.01 ‘**’, < 0.001 ‘***’). Previous success was coded as a factor (Success/Fail).

| **MODEL NUMBER** | **RESPONSE VARIABLE** | **MAXIMAL MODEL** | **FINAL MODEL** | **MODEL SELECTION** | **FINAL MODEL SUMMARY** | | | | | |
| --- | --- | --- | --- | --- | --- | --- | --- | --- | --- | --- |
|  |  |  |  |  |  | **Estimate** | **Std Error** | **df** | **T value** | **P value** |
| Mod13b | Δ Structural (g) | PreviousSuccess + (1\|MotherID) + (1\|Year) | PreviousSuccess + (1\|Year) | (1\|MotherID) explained 0 variance (Maximal model AIC: 546.6, Final model AIC: 544.6) | Intercept | -0.623 | 0.891 | 6.428 | -0.699 | 0.509 |
|  |  |  |  |  | Previous Success | 0.669 | 0.639 | 107.920 | 1.048 | 0.297 |
| Mod15b | Δ Structural (%) | PreviousSuccess + (1\|MotherID) + (1\|Year) | PreviousSuccess + (1\|Year) | (1\|MotherID) explained 0 variance (Maximal model AIC: 888.5, Final model AIC: 886.5) | Intercept | -10.500 | 3.211 | 14.718 | -3.270 | 0.005** |
|  |  |  |  |  | Previous Success | 12.616 | 3.077 | 108.922 | 4.101 | 7.96e-05*** |
| Mod17b | Δ Moss (g) | PreviousSuccess + (1\|MotherID) + (1\|Year) | PreviousSuccess + (1\|Year) | (1\|MotherID) explained 0 variance (Maximal model AIC: 529.8, Final model AIC: 527.8) | Intercept | -0.476 | 0.604 | 15.932 | -0.788 | 0.442 |
|  |  |  |  |  | Previous Success | 0.457 | 0.594 | 108.7 | 0.770 | 0.443 |
| Mod19b | Δ Moss (%) | PreviousSuccess + (1\|MotherID) + (1\|Year) | PreviousSuccess + (1\|Year) | (1\|MotherID) explained 0 variance (Maximal model AIC: 869.7, Final model AIC: 527.8) | Intercept | -0.476 | 0.604 | 15.932 | -0.788 | 0.442 |
|  |  |  |  |  | Previous Success | 0.487 | 0.594 | 108.706 | 0.770 | 0.443 |
| Mod21b | Δ Grass (g) | PreviousSuccess + (1\|MotherID) + (1\|Year) | PreviousSuccess + (1\|Year) | (1\|MotherID) explained 0 variance (Maximal model AIC: 466.4, Final model AIC: 464.4) | Intercept | -0.220 | 0.688 | 5.289 | -0.319 | 0.762 |
|  |  |  |  |  | Previous Success | 0.284 | 0.441 | 107.455 | 0.644 | 0.521 |
| Mod23b | Δ Grass (%) | PreviousSuccess + (1\|MotherID) + (1\|Year) | PreviousSuccess + (1\|Year) | (1\|MotherID) explained 0 variance (Maximal model AIC: 812.6, Final model AIC: 810.6) | Intercept | -3.540 | 2.750 | 7.452 | -1.287 | 0.237 |
|  |  |  |  |  | Previous Success | 4.808 | 2.168 | 108.335 | 2.218 | 0.029* |

**Table S5.** For significant models predicting a relationship between previous success and nest materials, we used the “emmeans” package to determine if each factor level was significantly different from zero (no change) between two years.

| **Model** | **Previous Success Level** | **emmean** | **SE** | **df** | **Lower CL** | **Upper CL** | **T ratio** | **P value** |
| --- | --- | --- | --- | --- | --- | --- | --- | --- |
| Mod15b | Fail | -10.50 | 3.26 | 13.89 | -17.51 | -3.49 | -3.217 | 0.006 |
|  | Success | 2.12 | 2.20 | 3.51 | -4.34 | 8.57 | 0.964 | 0.397 |

**Table S6.** Model structure, selection process, and summary output of general linear mixed models looking at the effect of previous success (count of previous fledglings) on the difference (Δ) in amount (g) and proportion (%) of structural material (moss and grass) included in nests between two consecutive attempts. All model output values are given to 3 decimal places. (Significance codes: < 0.05 ‘*’). Previous fledge was coded as numeric.

| **MODEL NUMBER** | **RESPONSE VARIABLE** | **MAXIMAL MODEL** | **FINAL MODEL** | **MODEL SELECTION** | **FINAL MODEL SUMMARY** | | | | | |
| --- | --- | --- | --- | --- | --- | --- | --- | --- | --- | --- |
|  |  |  |  |  |  | **Estimate** | **Std Error** | **df** | **T value** | **P value** |
| Mod14b | Δ Structural (g) | PreviousFledge + (1\|MotherID) + (1\|Year) | PreviousFledge + (1\|Year) | (1\|MotherID) explained 0 variance (Maximal model AIC: 439.2, Final model AIC: 237.2) | Intercept | 0.646 | 1.028 | 11.400 | 0.628 | 0.542 |
|  |  |  |  |  | Previous Fledge | -0.110 | 0.133 | 83.788 | -0.823 | 0.413 |
| Mod16b | Δ Structural (%) | PreviousFledge + (1\|MotherID) + (1\|Year) | PreviousFledge + (1\|Year) | (1\|MotherID) explained 0 variance (Maximal model AIC: 699.0, Final model AIC: 697.0) | Intercept | 2.554 | 4.034 | 25.146 | 0.633 | 0.532 |
|  |  |  |  |  | Previous Fledge | -0.081 | 0.618 | 84.938 | -0.131 | 0.896 |
| Mod18b | Δ Moss (g) | PreviousFledge + (1\|MotherID) + (1\|Year) | PreviousFledge + (1\|Year) | (1\|MotherID) explained 0 variance (Maximal model AIC: 427.3, Final model AIC: 425.3) | Intercept | 1.199 | 0.786 | 32.201 | 1.526 | 0.137 |
|  |  |  |  |  | Previous Fledge | -0.222 | 0.125 | 84.938 | -1.772 | 0.080 |
| Mod20b | Δ Moss (%) | PreviousFledge + (1\|MotherID) + (1\|Year) | PreviousFledge + (1\|Year) | (1\|MotherID) explained 0 variance (Maximal model AIC: 685.3, Final model AIC: 683.3) | Intercept | 2.411 | 3.788 | 21.186 | 0.363 | 0.531 |
|  |  |  |  |  | Previous Fledge | -0.282 | 0.570 | 84.814 | -0.495 | 0.622 |
| Mod22b | Δ Grass (g) | PreviousFledge + (1\|MotherID) + (1\|Year) | PreviousFledge + (1\|MotherID) + (1\|Year) | (1\|MotherID) explained 0 variance. However, examination of AIC suggests retaining (1\|MotherID) is the better model (Maximal model AIC: 371.6, removing (1\|MotherID) AIC: 466.6). Direction or significance of effects do not differ between models. | Intercept | -0.635 | 0.810 | 7.055 | -0.784 | 0.458 |
|  |  |  |  |  | Previous Fledge | 0.127 | 0.089 | 83.056 | 1.427 | 0.157 |
| Mod24b | Δ Grass (%) | PreviousFledge + (1\|MotherID) + (1\|Year) | PreviousFledge + (1\|Year) | (1\|Mother ID) explained very little variance (1.453e-06, sd = 0.001) (Maximal model AIC: 639.5, Final model AIC: 637.5) | Intercept | -0.375 | 3.383 | 10.595 | -0.111 | 0.914 |
|  |  |  |  |  | Previous Fledge | 0.292 | 0.433 | 83.675 | 0.676 | 0.501 |

**Table S7.** Model structure and summary outputs of hurdle models looking at the effects of Insulation: Structure ratio on hatching success, and fledging success of the clutch/brood raised in the nest. All model output values are given to 3 decimal places. (Significance codes: < 0.05 ‘*’, < 0.01 ‘**’, < 0.001 ‘***’). Insulation: Structure ratio is coded as numeric.

| **MODEL NUMBER** | **RESPONSE VARIABLE** | **MODEL STRUCTURE** | **MODEL SUMMARY** | | | | |
| --- | --- | --- | --- | --- | --- | --- | --- |
|  |  |  |  | **Estimate** | **Std Error** | **Z value** | **P value** |
| Mod25b | Hatchling Number | I:S ratio, dist = “negbin”, zero.dist = “binomial” | Count model (truncated negbin with log link) | | | | |
|  |  | | Intercept | 1.705 | 0.043 | 39.655 | < 2e-16 *** |
|  |  | | Insulation: Structure Ratio | 0.219 | 0.078 | 2.803 | 0.005 ** |
|  |  | | Log(theta) | 13.633 | 56.824 | 0.240 | 0.810 |
|  |  | | Zero hurdle model (binomial with logit link) | | | | |
|  |  | | Intercept | 1.049 | 0.278 | 3.782 | < 0.001 *** |
|  |  | | Insulation: Structure Ratio | 1.893 | 0.711 | 2.664 | 0.008 ** |
| Mod26b | Fledgling Number | I:S ratio, dist = “negbin”, zero.dist = “binomial” | Count model (truncated negbin with log link) | | | | |
|  |  | | Intercept | 1.533 | 0.052 | 29.475 | < 2e-16 *** |
|  |  | | Insulation: Structure Ratio | 0.274 | 0.089 | 3.092 | 0.002 ** |
|  |  | | Log(theta) | 9.268 | 51.162 | 0.181 | 0.856 |
|  |  | | Zero hurdle model (binomial with logit link) | | | | |
|  |  | | Intercept | -0.015 | 0.225 | -0.067 | 0.946 |
|  |  | | Insulation: Structure Ratio | 0.323 | 0.558 | 4.166 | 3.1e-05 *** |

**Table S8.** Model structure, selection process, and summary output of general linear mixed models looking at the effect of changes in the Insulation: Structure ratio (ΔI:S) nest structure on reproductive success. All model output values are given to 3 decimal places. (Significance codes: < 0.05 ‘*’, < 0.01 ‘**’, < 0.001 ‘***’). Insulation: Structure ratio is coded as numeric.

| **MODEL NUMBER** | **RESPONSE VARIABLE** | **MAXIMAL MODEL** | **FINAL MODEL** | **MODEL SELECTION** | **FINAL MODEL SUMMARY** | | | | | |
| --- | --- | --- | --- | --- | --- | --- | --- | --- | --- | --- |
|  |  |  |  |  |  | **Estimate** | **Std Error** | **df** | **T value** | **P value** |
| Mod27b | Δ Fledgling Number (all females) | ΔI:S + (1\|MotherID) + (1\|Year) | ΔI:S + (1\|Year) | (1\|MotherID) explained 0 variance (Maximal model AIC: 624.8, Final model AIC: 622.8) | Intercept | -0.404 | 0.858 | 3.038 | -0.471 | 0.670 |
|  |  |  |  |  | ΔI:S | 2.213 | 1.236 | 108.844 | 1.790 | 0.076 |
| Mod27d | Δ Fledgling Number (previously failed) | ΔI:S + (1\|MotherID) + (1\|Year) | ΔI:S + (1\|MotherID) | (1\|Year) explained 0 variance (Maximal model AIC: 119.6, Final model AIC: 117.6) | Intercept | 3.398 | 0.584 | 19.910 | 5.817 | 1.1e-05 *** |
|  |  |  |  |  | ΔI:S | 7.137 | 1.871 | 4.207 | 3.814 | 0.017 * |
| Mod27f | Δ Fledgling Number (previously success) | ΔI:S + (1\|MotherID) + (1\|Year) | ΔI:S + (1\|Year) | (1\|MotherID) explained 0 variance (Maximal model AIC: 477.4, Final model AIC: 475.4) | Intercept | -1.489 | 0.597 | 2.870 | -2.446 | 0.096 |
|  |  |  |  |  | ΔI:S | 0.064 | 1.254 | 75.862 | 0.051 | 0.960 |

**Table S9.** Model structure and summary outputs of hurdle models looking at the effects of reproductive success on heat retention (cooling coefficient), and general linear mixed models looking at the relationship between cooling coefficient and insulation. All model output values are given to 3 decimal places. (Significance codes: < 0.05 ‘*’, < 0.01 ‘**’, < 0.001 ‘***’). Cooling Coefficient and Insulation coded as numeric.

| **MODEL NUMBER** | | **RESPONSE VARIABLE** | | **MODEL STRUCTURE** | | | **MODEL SUMMARY** | | | | | | | | | | |
| --- | --- | --- | --- | --- | --- | --- | --- | --- | --- | --- | --- | --- | --- | --- | --- | --- | --- |
|  |  |  |  |  |  |  |  | | **Estimate** | | **Std Error** | | **Z value** | | | **P value** | |
| Mod28a | | Hatchling Number | | Cooling Coefficient, dist = “negbin”, zero.dist = “binomial” | | | Count model (truncated negbin with log link) | | | | | | | | | | |
|  | |  | | | | | Intercept | | 2.459 | | 0.127 | | 19.383 | | | < 2e-16 *** | |
|  | |  | | | | | Cooling Coefficient | | -24.906 | | 4.575 | | -5.444 | | | 5.22e-08 *** | |
|  | |  | | | | | Log(theta) | | 11.662 | | 23.823 | | 0.490 | | | 0.624 | |
|  | |  | | | | | Zero hurdle model (binomial with logit link) | | | | | | | | | | |
|  | |  | | | | | Intercept | | 5.339 | | 1.015 | | 5.258 | | | 1.46e-07 *** | |
|  | |  | | | | | Cooling Coefficient | | -104.780 | | 29.912 | | -3.503 | | | < 0.001 *** | |
| **MODEL NUMBER** | | **RESPONSE VARIABLE** | **MAXIMAL MODEL** | **FINAL MODEL** | **MODEL SELECTION** | **MODEL SUMMARY** | | | | | | | | | |  |  |
|  |  |  |  |  |  |  | **Estimate** | | **Std Error** | | **df** | | **T value** | **P value** | |  |  |
| Mod30a | | Cooling Coefficient | Insulation (g) + (1\|Year) + (1\|MotherID) | Insulation (g) + (1\|Year) + (1\|MotherID) | (1\|MotherID) explained 0 variance. However, examination of AIC suggests retaining (1\|MotherID) is the better model (Maximal model AIC: -1412.1, removing (1\|MotherID) AIC: -1406.4). Direction or significance of effects do not differ between models. | Intercept | 2.918e-02 | | 2.115e-03 | | 5.166e+00 | | 13.796 | 2.83e-05 *** | |  |  |
|  |  |  |  |  |  | Insulation | -2.703e-04 | | 1.852e-04 | | 1.907e+02 | | -1.459 | 0.146 | |  |  |
| Mod30c | | Cooling Coefficient | Insulation (%) + (1\|Year) + (1\|MotherID) | Insulation (%) + (1\|Year) + (1\|MotherID) | (1\|MotherID) explained 0 variance. However, examination of AIC suggests retaining (1\|MotherID) is the better model (Maximal model AIC: -1407.3, removing (1\|MotherID) AIC: -1401.96). Direction or significance of effects do not differ between models. | Intercept | 2.822e-02 | | 2.210e-03 | | 5.610e+00 | | 12.771 | 2.33e-05*** | |  |  |
|  |  |  |  |  |  | Insulation | -2.370e-06 | | 4.638e-05 | | 1.911e+02 | | -0.051 | 0.959 | |  |  |

**Table S10.** Model structure, selection process, and summary output of generalised linear mixed models (binomial) looking at the effect of personal experience on nestbox choice, and general linear model looking at the effect of nestbox choice on changes in fledgling number. All model output values are given to 3 decimal places. (Significance codes: < 0.05 ‘*’, < 0.01 ‘**’, < 0.001 ‘***’). All predictor variables are coded as numeric (New Male 1/0, Moved Nestbox 1/0).

| **MODEL NUMBER** | **RESPONSE VARIABLE** | **MAXIMAL MODEL** | **FINAL MODEL** | **MODEL SELECTION** | **FINAL MODEL SUMMARY** | | | | | | |
| --- | --- | --- | --- | --- | --- | --- | --- | --- | --- | --- | --- |
|  |  |  |  |  |  | **Estimate** | **Std Error** | **Z value** | **P value** | | |
| Mod31c | Moved Nestbox (1/0) | PreviousSuccess + (1\|MotherID) + (1\|Year) | PreviousSuccess + (1\|MotherID) | (1\|Year) explained almost 0 variance (1.408e-10, sd = 1.187e-05) (Maximal model AIC: 150.5, Final model AIC: 148.5) | Intercept | 1.223 | 0.647 | 1.89 | 0.059 | | |
|  |  |  |  |  | Previous Success | -1.386 | 0.711 | -1.95 | 0.051 | | |
| Mod32b | Moved Nestbox (1/0) | Previous Fledge + (1\|MotherID) + (1\|Year) | Previous Fledge + (1\|MotherID) | (1\|Year) explained almost 0 variance (3.487e-10, sd = 1.867e-05) (Maximal model AIC: 122.3, Final model AIC: 120.3) | Intercept | -0.605 | 0.686 | -0.881 | 0.378 | | |
|  |  |  |  |  | Previous Fledge | 0.080 | 0.113 | 0.708 | 0.479 | | |
| Mod34a | Moved Nestbox (1/0) | New Male (1/0) + (1\|MotherID) + (1\|Year) | New Male (1/0) + (1\|MotherID) + (1\|Year) | Both (1\|MotherID) and ((1\|Year) explained variation and the model fit retaining them both | Intercept | -10.534 | 2.898 | -3.653 | <0.001 *** | | |
|  |  |  |  |  | New Male | 21.654 | 4.998 | 4.333 | 1.47e-05 *** | | |
| **MODEL NUMBER** | **RESPONSE VARIABLE** | **MAXIMAL MODEL** | **FINAL MODEL** | **MODEL SELECTION** |  | **Estimate** | **Std Error** | **Df** | **T value** | **P value** | |
| Mod33b | Δ Fledgling Number (all females) | Moved Nestbox + (1\|MotherID) + (1\|Year) | Moved Nestbox + (1\|Year) | (1\|MotherID) explained 0 variance (Maximal model AIC: 628.6, Final model AIC: 626.6) | Intercept | -0.761 | 0.917 | 4.601 | -0.830 | | 0.447 |
|  |  |  |  |  | Moved Nestbox | 0.507 | 0.743 | 106.312 | 0.683 | | 0.496 |

**Proportional Success**

As we cannot be certain what a “successful” breeding attempt is for a female blue tit, we also ran analyses using proportion of eggs that survived to fledge (fledgling number/egg number) as our measure of reproductive success. We do not report this in the manuscript to avoid over-reporting measures of success, and because the measures reported (presence of a survived fledgling and number of survived fledglings) maximise information from our data. A previous survivorship analysis in this blue tit population has also identified one and seven fledglings as breakpoints in the distribution, verifying our use of fledgling presence (0/1) in our main analysis reported in the manuscript (Alexander *et al*., 2025).

Below we report the full model selection and output of models using proportional success to predict nest materials. Consistent with results from binary success, females with greater proportional success were more likely to reduce the mass of insulation in their subsequent nest, but this trend is insignificant. Again, this trend was driven by mass of mammal fur rather than feathers. Consistent with the results for binary success, females who had fledged a greater *proportion* of chicks, included a greater proportion, but not mass of structural material in their subsequent nest. However, with binary success this was driven by changes in grass, but for proportional success this was driven by changes in moss. The effect of nest changes on difference in proportional success also matched those for binary success. For females who previously failed to fledge any chicks, changing the nest design improved her proportional success, an effect we did not see for females who previously fledged at least one chick. Finally, contrasting with results for binary success, likelihood of moving nestbox did not depend on proportional success, and moving nestbox between years did not influence changes in proportional success.

**Table S11.** Model structure, selection process and summary output of general linear mixed models looking at the effect of previous success (proportion of eggs fledged) on the difference (Δ) in amount (g) and proportion (%) of insulation material (feathers and mammal hair) included in nests between two consecutive attempts. All model output values are given to 3 decimal places. (Significance codes: < 0.05 ‘*’).

| **MODEL NUMBER** | **RESPONSE VARIABLE** | **MAXIMAL MODEL** | **FINAL MODEL** | **MODEL SELECTION** | **FINAL MODEL SUMMARY** | | | | | | **χ^2^ OUTPUT** | |
| --- | --- | --- | --- | --- | --- | --- | --- | --- | --- | --- | --- | --- |
|  |  |  |  |  |  | **Estimate** | **Std Error** | **df** | **T value** | **P value** | **χ^2^ _df_** | **P Value** |
| Mod35b | Δ Insulation (g) | Previous Proportion + (1\|MotherID) + (1\|Year) | Previous Proportion + (1\|Year) | (1\|MotherID) explained 0 variance (Maximal model AIC: 511.2, Final model AIC: 509.2) | Intercept | 0.255 | 0.553 | 7.081 | 0.461 | 0.658 |  |  |
|  |  |  |  |  | Previous Proportion | -1.056 | 0.588 | 110.990 | -1.794 | 0.076 | 3.219_1_ | 0.073 |
| Mod36b | Δ Insulation (%) | Previous Proportion + (1\|MotherID) + (1\|Year) | Previous Proportion + (1\|Year) | (1\|MotherID) explained 0 variance (Maximal model AIC: 870.9, Final model AIC: 868.9) | Intercept | -1.153 | 3.327 | 5.263 | -0.346 | 0.742 |  |  |
|  |  |  |  |  | Previous Proportion | -1.279 | 2.971 | 110.609 | -0.430 | 0.668 | 0.185_1_ | 0.667 |
| Mod37b | Δ Mammal hair (g) | Previous Proportion + (1\|MotherID) + (1\|Year) | Previous Proportion + (1\|Year) | (1\|MotherID) explained 0 variance (Maximal model AIC: 494.6, Final model AIC: 492.6) | Intercept | 0.259 | 0.650 | 4.998 | 0.399 | 0.707 |  |  |
|  |  |  |  |  | Previous Proportion | -1.257 | 0.545 | 110.380 | -2.307 | 0.023* | 5.323_1_ | 0.021* |
| Mod38b | Δ Mammal hair (%) | Previous Proportion + (1\|MotherID) + (1\|Year) | Previous Proportion + (1\|Year) | (1\|MotherID) explained 0 variance (Maximal model AIC: 858.3, Final model AIC: 856.3) | Intercept | -0.245 | 3.497 | 4.727 | -0.07 | 0.947 |  |  |
|  |  |  |  |  | Previous Proportion | -3.446 | 2.803 | 110.202 | -1.23 | 0.221 | 1.512_1_ | 0.219 |
| Mod39b | Δ Feathers (g) | Previous Proportion + (1\|MotherID) + (1\|Year) | Previous Proportion + (1\|Year) | (1\|MotherID) explained some variance (0.046, sd = 0.214) Comparison of AIC suggested removing (1\|MotherID) was the better model (Maximal model AIC: 335.8, Final model AIC: 333.8). Keeping or removing (1\|MotherID) does not influence the direction or significance of results. | Intercept | 0.010 | 0.219 | 9.592 | 0.047 | 0.964 |  |  |
|  |  |  |  |  | Previous Proportion | 0.179 | 0.267 | 109.772 | 0.671 | 0.504 | 0.450_1_ | 0.502 |
| Mod40b | Δ Feathers (%) | Previous Proportion + (1\|MotherID) + (1\|Year) | Previous Proportion + (1\|Year) | (1\|MotherID) explained 0 variance (Maximal model AIC: 682.1, Final model AIC: 680.1) | Intercept | -0.988 | 0.977 | 11.052 | -1.011 | 0.334 |  |  |
|  |  |  |  |  | Previous Proportion | 2.288 | 1.264 | 107.486 | 1.810 | 0.073 | 3.276_1_ | 0.070 |

**Table S12.** Model structure, selection process, and summary output of general linear mixed models looking at the effect of previous success (proportion of eggs fledged) on the difference (Δ) in amount (g) and proportion (%) of structural material (moss and grass) included in nests between two consecutive attempts. All model output values are given to 3 decimal places. (Significance codes: < 0.05 ‘*’, < 0.01 ‘**’, < 0.001 ‘***’).

| **MODEL NUMBER** | **RESPONSE VARIABLE** | **MAXIMAL MODEL** | **FINAL MODEL** | **MODEL SELECTION** | **FINAL MODEL SUMMARY** | | | | | | **χ^2^ OUTPUT** | |
| --- | --- | --- | --- | --- | --- | --- | --- | --- | --- | --- | --- | --- |
|  |  |  |  |  |  | **Estimate** | **Std Error** | **df** | **T value** | **P value** | **χ^2^ _df_** | **P Value** |
| Mod41b | Δ Structural (g) | Previous Proportion + (1\|MotherID) + (1\|Year) | Previous Proportion + (1\|Year) | (1\|MotherID) explained 0 variance (Maximal model AIC: 557.4, Final model AIC: 555.4) | Intercept | -0.203 | 0.874 | 4.873 | -0.232 | 0.826 |  |  |
|  |  |  |  |  | Previous Proportion | 0.278 | 0.723 | 110.321 | 0.384 | 0.701 | 0.148_1_ | 0.701 |
| Mod42b | Δ Structural (%) | Previous Proportion + (1\|MotherID) + (1\|Year) | Previous Proportion + (1\|Year) | (1\|MotherID) explained 0 variance (Maximal model AIC: 914.0, Final model AIC: 912.0) | Intercept | -5.881 | 3.231 | 7.976 | -1.820 | 0.106 |  |  |
|  |  |  |  |  | Previous Proportion | 10.050 | 3.610 | 110.837 | 2.784 | 0.006 ** | 7.752_1_ | 0.005 ** |
| Mod43b | Δ Moss (g) | Previous Proportion + (1\|MotherID) + (1\|Year) | Previous Proportion + (1\|Year) | (1\|MotherID) explained 0 variance (Maximal model AIC: 538.2, Final model AIC: 536.2) | Intercept | -0.104 | 0.553 | 9.190 | -0.188 | 0.855 |  |  |
|  |  |  |  |  | Previous Proportion | 0.027 | 0.663 | 110.019 | 0.041 | 0.967 | 0.002_1_ | 0.967 |
| Mod44b | Δ Moss (%) | Previous Proportion + (1\|MotherID) + (1\|Year) | Previous Proportion + (1\|Year) | (1\|MotherID) explained 0 variance (Maximal model AIC: 887.7, Final model AIC: 885.7) | Intercept | -4.567 | 3.001 | 6.728 | -1.522 | 0.174 |  |  |
|  |  |  |  |  | Previous Proportion | 6.955 | 3.208 | 110.982 | 2.168 | 0.032* | 4.701_1_ | 0.030* |
| Mod45b | Δ Grass (g) | Previous Proportion + (1\|MotherID) + (1\|Year) | Previous Proportion + (1\|Year) | (1\|MotherID) explained 0 variance (Maximal model AIC: 887.7, Final model AIC: 885.7) | Intercept | -0.157 | 0.680 | 4.232 | -0.231 | 0.828 |  |  |
|  |  |  |  |  | Previous Proportion | 0.331 | 0.496 | 109.829 | 0.669 | 0.505 | 0.447_1_ | 0.504 |
| Mod46a | Δ Grass (%) | Previous Proportion + (1\|MotherID) + (1\|Year) | Previous Proportion + (1\|MotherID) + (1\|Year) | Both random effects explained variance so were retained (MotherID: 4.25, sd = 2.06, Year: 17.06, sd = 4.131) | Intercept | -1.480 | 2.646 | 5.272 | -0.559 | 0.599 |  |  |
|  |  |  |  |  | Previous Proportion | 3.282 | 2.475 | 105.287 | 1.326 | 0.188 | 1.758_1_ | 0.185 |

**Table S13.** Model structure, selection process, and summary output of general linear mixed models looking at the effect of changes in the Insulation: Structure ratio (ΔI:S) nest structure on changes in reproductive success (difference in proportion of eggs fledged). All model output values are given to 3 decimal places. (Significance codes: < 0.05 ‘*’, < 0.01 ‘**’, < 0.001 ‘***’).

| **MODEL NUMBER** | **RESPONSE VARIABLE** | **MAXIMAL MODEL** | **FINAL MODEL** | **MODEL SELECTION** | **FINAL MODEL SUMMARY** | | | | | | **χ^2^ OUTPUT** | |
| --- | --- | --- | --- | --- | --- | --- | --- | --- | --- | --- | --- | --- |
|  |  |  |  |  |  | **Estimate** | **Std Error** | **df** | **T value** | **P value** | **χ^2^ _df_** | **P Value** |
| Mod47b | Δ Fledgling Proportion (all females) | ΔI:S + (1\|MotherID) + (1\|Year) | ΔI:S + (1\|Year) | (1\|MotherID) explained 0 variance (Maximal model AIC: 183.2, Final model AIC: 181.2) | Intercept | 0.041 | 0.096 | 3.008 | 0.426 | 0.699 |  |  |
|  |  |  |  |  | ΔI:S | -0.221 | 0.160 | 109.583 | -1.379 | 0.717 | 1.901_1_ | 0.168 |
| Mod48c | Δ Fledgling Proportion (previously failed) | ΔI:S + (1\|MotherID) + (1\|Year) | ΔI:S + (1\|MotherID) | Model failed to converge with both random effects. (1\|Year) explained 0 variance. (1\|MotherID explained some variance (0.122, sd = 0.349). Comparison of AIC values suggests retaining (1\|MotherID) and removing (1\|Year) is the best model (Including (1\|MotherID) AIC: 22.6, retaining (1\|Year) AIC: 29.1). Retaining (1\|Year) or (1\|MotherID) does not influence the direction or significance of results. | Intercept | -0.464 | 0.075 | 20.669 | -6.148 | 4.54e-06 *** |  |  |
|  |  |  |  |  | ΔI:S | -1.127 | 0.096 | 2.110 | -11.726 | 0.006 ** | 137.5_1_ | <2.2e-16 *** |
| Mod49b | Δ Fledgling Proportion (previously success) | ΔI:S + (1\|MotherID) + (1\|Year) | ΔI:S + (1\|Year) | (1\|MotherID) explained 0 variance (Maximal model AIC: 125.6, Final model AIC: 123.6) | Intercept | 0.195 | 0.055 | 2.604 | 3.574 | 0.045 |  |  |
|  |  |  |  |  | ΔI:S | 0.043 | 0.152 | 50.530 | 0.285 | 0.777 | 0.082_1_ | 0.775 |

**Table S14.** Model structure, selection process, and summary output of generalised linear mixed models (binomial) looking at the effect of personal experience (proportion of eggs fledged) on nestbox choice, and general linear model looking at the effect of nestbox choice on changes in proportion of eggs fledged. All model output values are given to 3 decimal places. (Significance codes: < 0.05 ‘*’, < 0.01 ‘**’, < 0.001 ‘***’).

| **MODEL NUMBER** | **RESPONSE VARIABLE** | **MAXIMAL MODEL** | **FINAL MODEL** | **MODEL SELECTION** | **FINAL MODEL SUMMARY** | | | | | | **χ^2^ OUTPUT** | |
| --- | --- | --- | --- | --- | --- | --- | --- | --- | --- | --- | --- | --- |
|  |  |  |  |  |  | **Estimate** | **Std Error** | **Z value** | **P value** | | **χ^2^ _df_** | **P Value** |
| Mod50b | Moved Nestbox (1/0) | Previous Proportion + (1\|MotherID) + (1\|Year) | Previous Proportion + (1\|MotherID) | (1\|Year) explained 0 variance (Maximal model AIC: 157.2, Final model AIC: 155.2) | Intercept | 0.374 | 0.459 | 0.813 | 0.416 | |  |  |
|  |  |  |  |  | Previous Success | -0.043 | 0.668 | -0.649 | 0.516 | | 0.421_1_ | 0.516 |
| **MODEL NUMBER** | **RESPONSE VARIABLE** | **MAXIMAL MODEL** | **FINAL MODEL** | **MODEL SELECTION** | **FINAL MODEL SUMMARY** | | | | | | **χ^2^ OUTPUT** | |
|  |  |  |  |  |  | **Estimate** | **Std Error** | **Df** | **T value** | **P value** | **χ^2^ _df_** | **P Value** |
| Mod48b | Δ Fledgling Proportion | Moved Nestbox + (1\|MotherID) + (1\|Year) | Moved Nestbox + (1\|Year) | (1\|MotherID) explained 0 variance (Maximal model AIC: 186.0, Final model AIC: 184.0) | Intercept | 0.060 | 0.106 | 5.070 | 0.569 | 0.594 |  |  |
|  |  |  |  |  | Moved Nestbox | -0.020 | 0.096 | 108.360 | -0.212 | 0.833 | 0.045_1_ | 0.832 |

**References**

Alexander, E. J., Edwards, S. C., Chapman, E. G. & Healy, S. D. (2025). Previous reproductive success informs nest-building decisions. *Behavioural Ecology*, 36, araf009.
